# Anxiety among pregnant women during the first wave of the COVID-19 pandemic in Poland

**DOI:** 10.1038/s41598-022-12275-5

**Published:** 2022-05-19

**Authors:** Michalina Ilska, Anna Brandt-Salmeri, Anna Kołodziej-Zaleska, Heidi Preis, Emily Rehbein, Marci Lobel

**Affiliations:** 1 Institute of Psychology, University of Silesia in Katowice, Grażyńskiego Street 53, Katowice, Poland; 2 Department of Psychology, Stony Brook University, Stony Brook, NY 11794 USA

**Keywords:** Preventive medicine, Quality of life

## Abstract

Although anxiety is common because of the transitional nature of the perinatal period, particularly high levels of anxiety have been observed in some studies of pregnant women during the pandemic. The purpose of this study was to evaluate the severity of anxiety among pregnant women during the first wave of the COVID-19 pandemic in Poland, and factors associated with it. Cross-sectional study with a total of 1050 pregnant women recruited via social media in Poland during the first wave of the COVID-19 pandemic, from March 1 until June 1, 2020. The survey included validated psychological measures: the GAD-7 (anxiety), the PREPS (pandemic stress), with two subscales: preparedness and infection stress, and obstetric, sociodemographic and COVID-19 related variables. T-tests, ANOVAs, and hierarchical binary logistic regression for dichotomized GAD-7 scores (minimal or mild vs. moderate or severe) were used. Over a third of respondents experienced moderate or severe levels of anxiety. Predictors of moderate or severe anxiety were non-pandemic related factors like unplanned pregnancy and emotional and psychiatric problems, as well as pandemic related pregnancy stress. Levels of anxiety among pregnant women during the first wave of the COVID-19 pandemic in Poland exceeded pre-pandemic norms. Findings suggest that prior psychiatric conditions, unplanned pregnancy, and elevated pandemic-related pregnancy stress due to concerns about infection or poor preparation for birth contributed to the risk of high anxiety in Polish pregnant women during the pandemic onset. Given the harmful effects of antenatal anxiety on the health and well-being of mothers and their children, psychotherapeutic interventions, efforts to alleviate pregnant women’s stress, and training in adaptive ways to cope with stress are vital to reduce the prevalence of maternal anxiety and its potential consequences during this global crisis.

## Introduction

Before the COVID-19 pandemic, the World Health Organization reported that 10% of pregnant women and 13% of postpartum women experience a mental health disorder^1^. Of these, anxiety is a common disorder due to the transitional nature of the perinatal period^2,3^. Anxiety disorders can have deleterious consequences for pregnant women and their offspring. Antenatal anxiety is associated with increased risk of preterm delivery, reduced mother-infant bonding, and delays in cognitive/emotional development in infants, which can persist into childhood^4–6^. Thus, anxiety is an important mental health indicator in pregnancy, especially during the COVID-19 pandemic.

The COVID-19 pandemic has become a global health scourge with devastating consequences for society including widespread death, economic uncertainty, and strained health care systems^7^. It has triggered a wide variety of psychiatric problems for the general population, including panic disorder, anxiety, depression, and obsessive–compulsive disorder^8–10^. Travel restrictions, isolation, social distancing, limited access to medical care, and persistent daily life changes caused by the COVID-19 pandemic may increase the risk of mental health problems, especially in vulnerable populations such as pregnant women^11^, who face extra precautions^12,13^. Pregnancy itself can be stressful. Even in the absence of a pandemic, pregnant women commonly experience uncertainty about the future, concerns about their health and the health of their baby, and stress related to their imminent transition to motherhood^14^. The novelty and unpredictability imposed by the pandemic may intensify concerns and complicate the psychological well-being of pregnant women.

From mid-March until June 2020, the Polish government, like many other countries, imposed restrictions on movement but without total lockdown or travel bans^15^. Some restrictions affected the medical care of pregnant women^16,17^. For instance, many in-person antenatal care appointments were cancelled or converted to virtual appointments. Pregnant women dealt with prohibitions against hospital visits, delivery location changes due to closed clinics, and companions’ exclusion during labor and birth. Social distancing and extensive personal protective equipment used by medical staff often made childbirth an unnatural and cold experience. Moreover, based on early concerns about potential COVID-19 transmission, some mothers were separated from their babies in the immediate postnatal period and unable to breastfeed^18^. Although these measures were implemented to protect public health, there is evidence that such restrictions can have negative psychological effects on pregnant women^19^. Additionally, other conditions related to the pandemic—including exposure to interpersonal violence and economic hardship—may also elevate risk for perinatal psychiatric problems^20^. A growing number of studies have documented high levels of anxiety and depression in women pregnant during the pandemic^7,21,22^, but relatively few have distinguished the particular pandemic-related and pandemic-unrelated contributors to poor mental health, and none as of yet have reported on women in the country of Poland.

The purpose of this study was to (1) evaluate the severity of anxiety among pregnant women during the first wave of the COVID-19 pandemic in Poland; (2) verify the association of anxiety with sociodemographic factors, obstetric factors, COVID-19 related conditions, living situation, and two dimensions of pandemic-related pregnancy stress, preparedness stress and infection stress; (3) examine the extent to which pandemic-related conditions and stress are contributing to pregnant women’s anxiety over and above non-pandemic related factors that are also contributing to their anxiety.

## Methods

### Materials and procedures

The present study used a cross-sectional design to recruit 1050 pregnant women in Poland from March 1 until June 1, 2020, through social media (e.g., Facebook, pregnancy forums). The study questionnaire was completed through LimeSurvey, an online survey system. Inclusion criteria included: (1) pregnant, (2) at least 18 years old, (3) able to speak and understand Polish, (4) no COVID-19 diagnosis. Participants read the online consent form and provided informed consent prior to completing study instruments. The study was conducted in accordance with The Code of Ethics of the World Medical Association (Declaration of Helsinki). This study is part of a larger research project examining experiences of pregnant women during a pandemic. Ethical approval was provided by the Ethics Committee of Silesian University (approval no. KEUS.43/05.2020).

### Measures

#### Anxiety

*The Generalized Anxiety Disorder-7* (GAD-7)^23^ is a validated 7-item self-report instrument that assesses the severity of generalized anxiety symptoms. Respondents report the frequency of anxiety symptoms over the past two weeks on a scale ranging from 0 = Not at All to 3 = Nearly Every Day. Individual scores were calculated as a sum of item responses ranging from 0 to 21. In addition, scores of 0–9 and 10–21 were used as cut-offs for minimal and mild vs. moderate and severe anxiety, respectively, as recommended^23^. The scale was internally consistent, Cronbach’s alpha = 0.92.

#### COVID-19 related stress

*The Pandemic-Related Pregnancy Stress Scale* (PREPS)^24^ is a novel instrument that assesses prenatal stress during a pandemic. It performed well psychometrically in this study, as reported previously^25^. The PREPS includes a subscale that assesses stress about preparation for birth and the postpartum (PREPS-Preparedness; PREPS-PS), and a second subscale that assesses stress involving concerns about infection of oneself or one’s fetus/baby (PREPS-Infection; PREPS-IS). Both scales were internally consistent (PREPS-PS α = 0.83; PREPS-IS α = 0.88). A third PREPS subscale assessing positive appraisal was not pertinent to this study and therefore not used. Items are rated on a scale from 1 = Very Little to 5 = Very Much. Scores were calculated as the mean response of items on the corresponding subscale (range 1 to 5).

Sociodemographic characteristics included maternal age (coded younger < 35/older ≥ 35), financial status (below average/ average/ above average), relationship status (some or no relationship/married or cohabiting).

Obstetric factors included multiparous (no/yes), gestational age (in weeks and coded by trimester), high risk pregnancy (no/yes/unsure), chronic medical conditions (no/yes), unplanned pregnancy (no/yes), and fertility treatments (no/yes).

COVID-19 related conditions (all no/yes) included loss of income because of COVID-19, obstetric visit cancelled or rescheduled because of COVID-19, suspected COVID-19 infection without being medically diagnosed, and access to outdoor space.

Other predictors. Because of their likely association with anxiety, three additional factors were assessed with dichotomous questions (no/yes): major life event while pregnant, experience of lifetime abuse, and current emotional or psychiatric problems.

### Statistical analysis

In the first stage of statistical analysis, mean differences in the continuous GAD-7 anxiety score for women with different sociodemographic characteristics, obstetric factors, COVID-19 related conditions, and other predictors were evaluated using Independent Sample *t*-tests with Cohen’s d effect size, or ANOVA and eta squared effect size, as appropriate. Moreover, associations of the GAD-7 score with PREPS-IS and PREPS-PS were examined with Pearson’s correlations.

Following this stage, all variables that exhibited significant associations with the continuous GAD-7 anxiety score in bivariate analyses were entered into a hierarchical binary logistic regression model to predict odds risk for minimal or mild anxiety (GAD-7 = 0–9) versus moderate or severe anxiety (GAD-7 = 10–21). The predictors of moderate or severe anxiety in Step 1 included the non-COVID-19 related variables: financial status, lifetime abuse, current emotional or psychiatric problems, major life event, unplanned pregnancy, high risk pregnancy, and chronic medical conditions. Step 2 included COVID-19 related variables: loss of income because of COVID-19, obstetric visit canceled or rescheduled, access to outdoor space, pandemic-related stress in two dimensions: infection and preparedness. The criterion for statistical significance was *p* < 0.05 for all analyses.

## Results

Participants’ average age was 30.48 ± 4.01 years old and their average gestational age was 27 (M = 26.99; SD = 9.19) weeks. Nearly half were nulliparas (n = 455, 43.4%). Other participant characteristics are displayed in Table 1. High rates of anxiety were observed in the sample: 368 participants (35%) reported mild anxiety symptoms (GAD-7 = 5–9), 172 (21.6%) reported moderate anxiety symptoms (GAD-7 = 10–14), and 122 (11.6%) reported severe anxiety symptoms (GAD-7 ≥ 15). Approximately a quarter (25.4%) and more than a third (36.5%) of women scored a 4 or higher on the PREPS-IS subscale and PREPS-PS subscale, respectively, indicating high levels of COVID-19 related pregnancy stress.

**Table 1:** Sample characteristics and mean differences in GAD-7 scale score based on sociodemographic characteristics, obstetric factors, and other predictors (N = 1050).

	N (%)	GAD-7
**Sociodemographic characteristics**		
***Age (year)***		*t* = −1.34
Younger (< 35)	894 (85.1)	7.19 ± 5.39
Older (≥ 35)	156 (14.9)	6.62 ± 4.81
***Relationship status***		*t* = −0.97
Some or no relationship	32 (3.0)	6.21 ± 5.52
Married or cohabiting	1014 (96.6)	7.14 ± 5.29
***Financial status***		***F*** = 7.17***; η^**2**^ **= 0.01**
Below average	69 (6.6)	9.13 ± 5.89^a^
Average	669 (63.7)	7.18 ± 5.21^b^
Above average	312 (29.7)	6.50 ± 5.26^b^
**Other variables**		
***Lifetime abuse***		*t* = 3.66***; ***d* = 0.44**
Yes	64 (6.1)	9.40 ± 5.76
No	986 (93.9)	6.95 ± 5.24
***Current emotional or psychiatric problems***		***t*** = 9.87***; ***d* = 1.03**
Yes	101 (9.6)	12.01 ± 5.37
No	949 (90.4)	6.58 ± 5.03
***Major life event while pregnant***		*t* = 3.03**; ***d* = 0.23**
Yes	266 (25.3)	8.01 ± 5.53
No	784 (74.7)	6.76 ± 5.19
**Obstetric factors**		
***Unplanned pregnancy***		***t*** = −3.01**; ***d* = 0.25**
Yes	160 (15.2)	8.26 ± 5.49
No	890 (84.8)	6.90 ± 5.25
***First child***		*t* = −1.48
Yes	455 (43.4)	6.83 ± 5.32
No	594 (56.6)	7.32 ± 5.30
***Trimester***		*F* = 2.38
1st	111 (10.6)	6.59 ± 4.71
2nd	344 (32.8)	6.74 ± 5.14
3rd	594 (56.6)	7.42 ± 5.49
***High risk pregnancy***		*F* = 8.10***; η ^2^** = 0.015**
Yes	119 (11.3)	8.89 ± 6.17^a^
No	882 (84.0)	6.84 ± 5.17^b^
Unsure	49 (4.7)	7.51 ± 4.74^ab^
***Chronic medical conditions***		*F* = 9.32***; η^**2**^ = **0.018**
Yes	295 (28.1)	8.01 ± 5.45^a^
No	744 (70.9)	6.69 ± 5.18^b^
Unsure	11 (1.0)	10.81 ± 5.82^a^
***Fertility treatments***		*t* = 0.33
Yes	86 (8.2)	7.29 ± 5.31
No	963 (91.7)	7.08 ± 5.31

*p < *.*05; **p < *.*01; ***p < *.*001.

Estimates of effect sizes: d—Cohen’s d; η^2^—eta squared.

Means with different superscripts are significantly different at *p* < 0.05 in a post hoc Scheffé test.

In bivariate analyses, anxiety was related to financial status, lifetime abuse, current emotional or psychiatric problems, and having experienced a major life event during pregnancy. Likewise, anxiety was associated with most of the obstetric factors including unplanned pregnancy, high risk pregnancy, and chronic medical conditions (see Table 1). As shown in Table 2, anxiety was also associated with all but one of the COVID-19 related variables.

**Table 2:** Sample characteristics and mean differences in GAD-7 scale score based on COVID-19 related conditions (N = 1050).

COVID-19 related conditions	N (%)	GAD-7
**Loss of income because of COVID-19**		*t* = 2.85**; ***d* = 0.20**
Yes	297 (28.3)	7.87 ± 5.58
No	753 (71.7)	6.80 ± 5.16
**Obstetric visit cancelled or rescheduled**		***t*** = 2.48*; ***d* = 0.16**
Yes	343 (32.7)	7.69 ± 5.45
No	707 (67.3)	6.82 ± 5.22
**Suspected COVID-19 infection**		*F* = 1.44
Yes	52 (5.0)	7.46 ± 5.13
No	718 (68.4)	6.91 ± 5.33
Unsure	280 (26.7)	7.52 ± 5.25
**Access to outdoor space**		*F* = 20.50***; η ^**2**^** = 0.038**
Yes, whenever I want	743 (70.8)	6.52 ± 5.14^a^
Sometimes	220 (21.0)	7.95 ± 5.21^b^
Rarely	87 (8.3)	9.95 ± 5.81^c^

*p < *.*05; **p < *.*01; ***p < *.*001.

Estimates of effect sizes: d—Cohen’s d; η^2^—eta squared.

Means with different superscripts are significantly different at *p* < 0.05 in a post hoc Scheffé test.

Descriptive statistics and correlations among anxiety and PREPS subscale scores are presented in Table 3. Anxiety was positively correlated both with PREPS-IS and with PREPS-PS (see Table 3).

**Table 3:** Means, standard deviations, and intercorrelations among COVID-19 related stress and anxiety (*N* = 1050)*.*

	*M*	*SD*	PREPS-IS	PREPS-PS
PREPS-IS	2.98	1.14		
PREPS-PS	3.46	0.95	.582**	
GAD-7	7.10	5.31	.355**	.446**

*PREPS-IS* pandemic-related pregnancy infection stress, *PREPS-PS* pandemic-related pregnancy preparedness stress, *GAD-7* generalized anxiety disorder.

**p < .01 *p < .05.

### Multivariate analyses

Hierarchical logistic regression analysis was carried out to calculate the adjusted odds ratio (AOR) for those who reported the highest level of anxiety symptoms (GAD-7 ≥ 10). As shown in Table 4, the model to predict moderate or severe anxiety used variables that exhibited significant bivariate associations with the continuous GAD-7 score, including non-COVID-19 related variables: financial status, lifetime abuse, current emotional or psychiatric problems, major prenatal life event, unplanned pregnancy, high risk pregnancy, and chronic medical conditions; and COVID-19 related variables: loss of income, obstetric visit cancelled or rescheduled, access to outdoor space, pandemic-related infection and preparedness stress (PREPS-IS, PREPS-PS).

**Table 4:** Binary hierarchical logistic regression predicting moderate or severe anxiety symptoms (*N* = 1050).

	Step 1		Step 2	
	AOR	95% CI	AOR	95% CI
Financial status^†^	1.25	(0.91, 1.73)	1.13	(0.79, 1.60)
Lifetime abuse	1.63	(0.93, 2.85)	1.30	(0.70, 2.40)
Emotional or psychiatric problems	4.88***	(3.09, 7.68)	5.16***	(3.12, 8.54)
Major life event	1.34	(0.97, 1.84)	1.39	(0.98, 1.97)
Unplanned pregnancy	1.67*	(1.15, 2.43)	2.37***	(1.57, 3.60)
High risk^†^	1.35	(0.92, 1.97)	1.23	(0.81, 1.87)
Chronic illness^†^	1.51*	(1.10, 2.06)	1.25	(0.88, 1.76)
Income lost			1.12	(0.80, 1.58)
Appointment altered			1.17	(0.85, 1.63)
Limited access to outdoor space^†^			1.24	(0.89, 1.73)
PREPS—infection			1.06**	(1.02, 1.09)
PREPS—preparedness			1.13***	(1.09, 1.17)
	*R*^*2*^ = 0.13		*R*^*2*^ = 0.31	

*AOR* adjusted odds ratio, *CI* confidence interval.

*p < 0.05, **p < 0.01, ***p < 0.001.

^†^Women who reported below average or average financial status were grouped together; Women who reported sometimes or rarely having access to outdoor space were grouped together; Women who reported having a chronic illness and those who were unsure were grouped together; Women who reported being high risk and those who were unsure were grouped together.

The first step of the regression included non-COVID-19 related variables which predicted 13% of the variance in anxiety symptoms with current emotional or psychiatric problems, chronic illness, and unplanned pregnancy uniquely increasing the odds of moderate to severe symptoms. In the second step, COVID-19 related variables increased the explained variance by an additional 18%. In the final model (total *R*^2^ = 0.31), current emotional or psychiatric problems (AOR 5.16, *p* < 0.001), unplanned pregnancy (AOR 2.37, *p* < 0.001), PREPS Infection Stress (AOR 1.06, *p* < 0.01), and PREPS Preparedness Stress (AOR 1.13, *p* < 0.001) all independently predicted greater likelihood of moderate or severe anxiety. Chronic illness was no longer significant in the final model.

## Discussion

### Main findings

Our research documents elevated anxiety among Polish pregnant women during the first wave of the COVID-19 pandemic: over a third of respondents experienced moderate to severe levels of anxiety.

Several groups of women reported more frequent anxiety symptoms. Bivariate analyses identified a number of factors associated with anxiety, but not all of these were predictive when examined simultaneously in the multivariate analysis, suggesting overlap among some of the predictors. When predictors were examined simultaneously in multivariate analyses, greater risk for moderate or severe generalized anxiety symptoms was evident for pregnant women who had experienced psychiatric problems, those with an unplanned pregnancy, and women who were experiencing significant stress related to their concerns about being prepared to give birth during the pandemic or because they were worried about the risk of infection to themselves or their baby. Notably, a quarter of participants experienced high levels of COVID-19 related pregnancy infection stress and more than a third exhibited high levels of COVID-19 related preparedness stress. These results are consistent with findings reported in a study of pandemic-related stress and anxiety among pregnant women in the US^22^ and with other research documenting the impact of COVID-19 distress on mental health^26^.

Two additional factors were found to be independent predictors of moderate or severe anxiety in this study. One factor, having an unplanned pregnancy, corroborates the results of other studies linking unplanned pregnancy to anxiety and depression (for review see ^2^). Emotional distress related to an unplanned and possibly unwanted pregnancy may worsen along with the uncertainty of a pandemic and become fertile ground for anxiety disorders, particularly in Poland where access to contraception is limited and abortion has recently become illegal. A second factor independently predicting high anxiety, as might be expected, was reporting that one has a current emotional or psychiatric problem. A review conducted by Biaggi et al.^2^ demonstrated that current or previous history of psychiatric illness is a risk factor for the development of antenatal anxiety. Additionally, general population studies from around the world have established that current or previous history of psychiatric illness is associated with negative mental health consequences of COVID-19^27–29^. Individuals with psychiatric illness are likely to be more susceptible and sensitive to the effects of pandemic-related stress, and given the restrictions in place at many health care settings with a simultaneous increase in demands for them, those in need may face difficulties obtaining psychiatric services^30^.

### Implications

The pandemic has led to a sizeable increase in the percentage of pregnant women displaying symptoms of anxiety. In our sample of Polish women, levels of general anxiety during the first wave of the pandemic exceeded pre-pandemic norms in pregnancy. According to a recent meta-analysis, the pre-pandemic prevalence of any anxiety disorder was 15.2% and 22.9% for self-reported anxiety symptoms^3^, considerably lower than the two-thirds of our sample (68.2%) who reported mild, moderate, or severe levels and still lower than the one third (33.2%) who reported moderate or severe anxiety. Our findings are in line with other research conducted during the COVID-19 pandemic that shows high levels of anxiety in pregnant women from different nations and geographic, cultural, or sociopolitical backgrounds, including Canada, the US, the United Kingdom, Australia, India, Israel, and China^22,31–33^. As shown in the current study, anxiety is associated with stress related to fears of infection and feeling unprepared for childbirth due to pandemic restrictions. Thus, the pervasiveness of high anxiety among pregnant women across the world likely reflects similar concerns for their health and survival and that of their child, as well as stress originating from the widespread limitations imposed on usual practices for giving birth.

Our findings point to factors that may help in identifying pregnant women who are at risk of experiencing high anxiety during this pandemic and possibly, during future crises. Understanding key risk factors may help health care professionals to intervene earlier, potentially preventing the progression of anxiety developing into full-blown disorder and reducing its cumulative impact on women and their offspring. Pregnant women with current emotional or psychiatric problems, those with an unplanned pregnancy, and women who are experiencing high stress appear to be at greater risk^24,34^. In addition to appropriate psychotherapeutic interventions, efforts to alleviate pregnant women’s stress related to preparation for birth and concerns about infection, as well as offering training in adaptive ways to cope with such stress are likely to reduce the prevalence of antenatal anxiety. Given evidence of the harmful effects of anxiety during pregnancy on the health and well-being of mothers and their children^4–6^, including effects that can be long-lasting, identification and intervention with women at risk are vital, especially during a pandemic that has gripped the globe and elevated anxiety for many pregnant women in its midst.

### Strengths and limitations

A strength of this study lies in its large sample of Polish pregnant women during the first wave of COVID-19, with data collected in close temporal proximity to the World Health Organization announcement of the COVID-19 pandemic. In addition, to the best of our knowledge, this is the first study in Poland that has investigated the prevalence of factors associated with mental health symptoms in pregnant women using standardized rating scales during the COVID-19 pandemic.

The present research has some limitations, too. The sample consisted mostly of young, married, or cohabiting women with average or good financial status. Another constraint was the exclusion of women known to be infected by COVID-19, which was deemed appropriate because the study was conducted at a time when there were few diagnosed cases of the disease among pregnant women in Poland. The use of an online survey also limited the sample to women with access to an Internet-enabled device. These study features reduce the generalizability of results. Furthermore, due to the cross-sectional design of this study, we cannot ascertain cause and effect. Longitudinal research is needed to verify the trajectory and mediators of these study findings. It is unclear whether the pandemic has caused or exacerbated maternal mental health problems or whether some intermediary processes exist that have influenced pregnant women’s mental health. Data collected with interview-based assessments and medical chart data will be valuable to replicate and extend these findings.

## Conclusions

The COVID-19 pandemic has contributed to poorer mental health in pregnant women around the world. Results of this study indicate that anxiety is prevalent among pregnant women in Poland. Among Polish pregnant women, those with an unplanned pregnancy, those with other emotional or psychiatric problems, and women experiencing high pandemic-related stress are at elevated risk of moderate or severe anxiety. Interventions to alleviate maternal anxiety can benefit mothers and children now and long beyond the current pandemic.
